# Forensic age estimation via magnetic resonance imaging of knee in the Turkish population: use of T1-TSE sequence

**DOI:** 10.1007/s00414-020-02402-0

**Published:** 2020-08-24

**Authors:** Oguzhan Ekizoglu, Ali Er, Mustafa Bozdag, Can Doruk Basa, Ismail Eralp Kacmaz, Negahnaz Moghaddam, Silke Grabherr

**Affiliations:** 1 Department of Forensic Medicine, Tepecik Training and Research Hospital, Güney mahallesi 1140/1 Yenisehir, Konak, Izmir, Turkey; 2 University Center of Legal Medicine, Lausanne - Geneva, Switzerland; 3 Department of Radiology, Tepecik Training and Research Hospital, Izmir, Turkey; 4 Department of Orthopaedics, Tepecik Training and Research Hospital, Izmir, Turkey; 5 Unit of Forensic Imaging and Anthropology, University Center of Legal Medicine, Lausanne - Geneva, Switzerland; 6 Swiss Human Institute of Forensic Taphonomy, University Center of Legal Medicine , Lausanne - Geneva, Switzerland

**Keywords:** Age estimation, Distal femoral epiphysis, Proximal tibial epiphysis, Magnetic resonance imaging

## Abstract

The evaluation of epiphyseal areas by magnetic resonance imaging (MRI) for forensic age estimation is an important supportive diagnostic method to prevent repeated radiation exposure without a valid medical reason. There are still not enough individuals being analyzed with MRI for age estimation. The aim of this study was to investigate the utility of T1-weighted turbo spin echo (T1-TSE) MRI sequences in determining the degree of ossification of the distal femoral and proximal tibial epiphyses in a Turkish population. In this study, images from 649 patients (335 males and 314 females) aged 10–30 years were retrospectively evaluated with sagittal T1-weighted turbo spin echo (T1-TSE) MRI sequences of the knee. Proximal tibial and distal femoral epiphysis were scored by two different observers twice using the combined staging system described by Schmeling and Kellinghaus. Spearman’s rank correlation analysis indicated a significant positive relationship between age and ossification stages of the distal femoral and proximal tibial epiphyses (*p* < 0.001). The intra- and inter-observer reliabilities in evaluating the femur and tibia were separately determined and gave promising results and Cohen’s kappa statistics ranged from κ = 0.886 and κ = 0.961. The minimal ages of patients with stage 4 ossification were 15.1 years for females and 15.8 years for males for the distal tibial epiphysis and 15.4 years for females and 17 years for males for the distal femoral epiphysis. This study show that (T1-TSE) MRI and the applicability and Schmeling and Kellinghaus staging method of the knee can be performed for living 14- to 17-year-old individuals in need of a supportive noninvasive method for estimating forensic age.

## Introduction

Failure to submit a valid birth registration to legal authorities remains a problem for underdeveloped countries as well as a legal problem for immigrants from these and developed countries [1]. Forensic age estimation plays a decisive role in many criminal and legal topics. It is important to determine the methodology to be used in the provision of detention conditions for children, requests for asylum, human trafficking, child adoption, child abuse and marriage, and forensic medical examinations and the subsequent reporting for determining the main criminal liability [2–8]. Especially in the implementation of the laws and civil rights specific to children and young adults, age limits should be estimated, with the primary goal of estimating the possible minimum age of individuals in the 14–21 age group [4–7].

In forensic medical evaluations, the approach recommended by the “Forensic Age Diagnostics of the German Society of Legal Medicine” is a “combination of a physical and radiographic examination of the left hand, a dental examination, and an orthopantomographic examination. If ossification of the hand is complete, radiological examination of the degree of clavicular ossification with conventional radiography and/or computed tomography (CT) is recommended” [5]. The combined use of methods can be important to achieve results with minimal errors. In legal proceedings where legal age should be estimated, the “minimum age concept” [4] is applied, and in cases where supportive data are needed to estimate the age, the minimum age limits obtained by MRI studies can be used as a guide. Researchers have suggested noninvasive methods, especially considering the ethical concerns posed by repeated radiation exposure in the pediatric age group [9–11]. Although an increasing number of studies have been published in recent years, especially for MR-based methods, such noninvasive methods have not yet been included in the AGFAD (Study Group on Forensic Age Diagnostics of the German Association of Forensic Medicine) proposal. This can be explained by the lack of an adequate and comparative database.

Assessments of different sequences for detecting ossification of the distal femoral epiphysis and proximal tibial epiphysis have been made with MRI, and past studies have shown that both ossification centers, especially the distal femoral epiphysis, can offer minimal age limits that can be valuable for forensic age estimation [12–20].

The system defined by Schmeling et al. [21] and Kellinghaus et al. [22], based on the anatomical staging of epiphyseal development, can be easily implemented with T1-weighted MRI sequences. In past T1-weighted MRI studies, on the evaluation of the distal femoral epiphysis [15–17], proximal tibial epiphysis [14, 16, 17, 20], distal radial epiphysis [23, 24], and proximal humeral epiphysis [25], the anatomical structure was successfully investigated by using the staging system defined by Schmeling et al. [21] and Kellinghaus et al. [22]. The results showed that the data obtained by MRI of the knee region is a potential supportive age estimation method for German and Chinese population [14–17, 20]. In this study, to investigate whether the examination of the distal femoral and proximal tibial epiphysis with T1-TSE sequence MRI, which is the closest depiction of bone, will reliability and validity to the age assessment in Turkish population and contributes to the age estimation database made with staging method of Schmeling et al. [21] and Kellinghaus et al. [22] using T1-TSE sequence MRI.

## Material and methods

This study was conducted at the Izmir Tepecik Training and Research Hospital, Turkey. The clinical and radiological data of patients admitted to the various clinics of the hospital from 2016 to 2019 with diagnoses of trauma and knee pain were retrospectively evaluated. The study protocol was approved by the hospital’s ethics committee. After we examined the medical records and MRI scans, socioeconomic status and ethnicity information were not included in the medical records; therefore no assessment has been made on this subject. Otherwise patients with any pathology of the knee (e.g., tumor, fracture, infection, surgical fixation, or bone marrow edema), patients with neoplastic disorders, patients undergoing radiotherapy or chemotherapy, and patients with MRI scans with motion artifacts were excluded. A total of 67 patients with fracture (41), bone marrow edema (8), surgical fixation (5), hypothyroidism (2), and MRI scans with motion artifacts (11) were excluded from the study.

Finally, we obtained data for 649 patients (335 males and 314 females) aged 10–30 years. The MRI of the left knee was conducted using a Siemens Magnetom Aera 1.5 T machine (Siemens Medical Systems, Erlangen, Germany) and a knee coil. For the analysis, T1-weighted turbo spin echo (T1-TSE) sequences in the sagittal plane were used. The imaging parameters were as follows: TR, 345 ms; TE, 11 ms; matrix, 512 × 512; FOV, 180 mm; and slice thickness, 1.5 mm. A workstation (Syngo; Siemens Medical Systems) with a high-resolution diagnostic monitor was used to evaluate the MRI scans.

Two observers (R1 and R2) evaluated all MR images. One experienced expert in legal medicine (R1) and one experienced radiologist (R2) evaluated each MRI scan twice. The observers re-evaluated all images after 4 weeks without knowledge of the results of the previous staging. We analyzed the proximal tibial and distal femoral epiphyses using the combined scoring system described by Schmeling et al. [21] and Kellinghaus et al. [22] as shown in Table 1.

**Table 1:** Combined scoring system described by Schmeling et al. [21] and Kellinghaus et al. [22]

Stage	Description
Stage 1	Ossification center is not yet ossified
Stage 2	Ossification center is ossified. Epiphyseal cartilage is not yet ossified
Stage 2a	The lengthwise epiphyseal measurement is one third or less of the widthwise measurement of the metaphyseal ending
Stage 2b	The lengthwise epiphyseal measurement is more than one third to two thirds of the widthwise measurement of the metaphyseal ending
Stage 2c	The lengthwise epiphyseal measurement is over two thirds of the widthwise measurement of the metaphyseal ending
Stage 3	Epiphyseal cartilage is partially ossified
Stage 3a	Epiphyseal-metaphyseal fusion completes one third or less of the former gap between the epiphysis and metaphysis.
Stage 3b	Epiphyseal-metaphyseal fusion completes over one third to two thirds of the former gap between the epiphysis and metaphysis.
Stage 3c	Epiphyseal-metaphyseal fusion completes over two thirds of the former gap between the epiphysis and metaphysis.
Stage 4	Epiphyseal cartilage is completely ossified. Epiphyseal scar is visible
Stage 5	Epiphyseal cartilage is completely ossified. Epiphyseal scar is no longer visible

### Statistical analyses

Statistical Package for the Social Sciences (SPSS) software (ver. 17; IBM Corporation, Armonk, NY, USA) was used for the statistical analyses. Data were expressed as the means or medians, with standard deviations (SDs), lower and upper quartiles, and minimum and maximum values, as appropriate. Associations between age and ossification stage were evaluated using Spearman’s correlation analysis. Between-sex comparisons were made using the Mann-Whitney U test. A *p* value < 0.01 was taken to reflect statistical significance.

The extent of agreement between observers and time points was assessed using Cohen’s κ test. The κ values, weighted κ values, and agreement rates were calculated. The system developed by Altman [26] was used to interpret the κ values.

## Results

In this study, images from 649 patients (335 males and 314 females) aged 10–30 years were evaluated (Table 2). The mean ages of the male and female patients were 20.56 ± 5.11 and 20.26 ± 5.77 years, respectively. Intra- and inter-observer agreements in evaluating the distal femoral and proximal tibial epiphysis were separately calculated. The intra-observer agreement for the distal femoral epiphysis was κ =0.924, and the inter-observer reliability was κ =0.898. The intra-observer agreement for the proximal tibial epiphysis was κ =0.961, and the inter-observer reliability was κ =0.886. Thus, intra-observer and inter-observer evaluation showed very good repeatability and consistency of the method for both the distal femoral and proximal tibial epiphysis. Spearman’s rank correlation analysis indicated a significant positive relationship between age and the stage of ossification of both epiphyses (distal femoral epiphysis: all subjects: rho = 0.739, *p* < 0.001; males: rho = 0.744, *p* < 0.001; females: rho = 0.735, *p* < 0.001; proximal tibial epiphysis: all subjects: rho = 0.704, *p* < 0.001; males: rho = 0.709, *p* < 0.001; females: rho = 0.704, *p* < 0.001). Statistical analysis of sex-related differences was performed; for the distal femoral epiphysis, significant differences were found for stage 2c (*p* < 0.05), stage 3a (*p* < 0.01), and stage 3b (p < 0.05) but not for stage 3c (*p* = 0.06) and stage 4 (*p* = 0.610), while for the proximal tibial epiphysis, significant differences were found for stage 3a (*p* < 0.01) and stage 3c (*p* < 0.01), but not for stage 2c (*p* = 0.106), stage 3b (*p* = 0.4), and stage 4 (*p* = 0.44).

**Table 2:** Age distribution of male and female subjects

Age (years)	Male (*N*)	Female (*N*)
10	10	12
11	4	20
12	6	6
13	20	14
14	6	10
15	36	14
16	24	10
17	21	22
18	12	15
19	17	15
20	19	23
21	11	9
22	25	14
23	19	11
24	18	22
25	21	15
26	23	23
27	17	11
28	18	22
29	6	20
Total	335	314

**Fig. 1:**
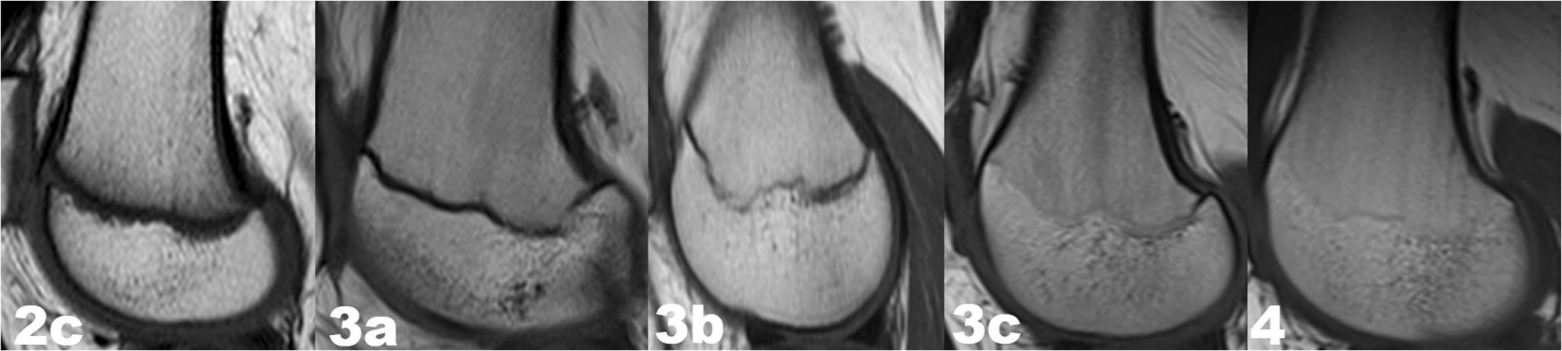
T1-weighted turbo spin echo (T1-TSE) sequences in the sagittal plane on MRI images: stages 2c,3a,3b,3c, and 4 according to combined scoring system described by Schmeling et al. [21] and Kellinghaus et al. [22] for distal femoral epiphysis

**Fig. 2:**
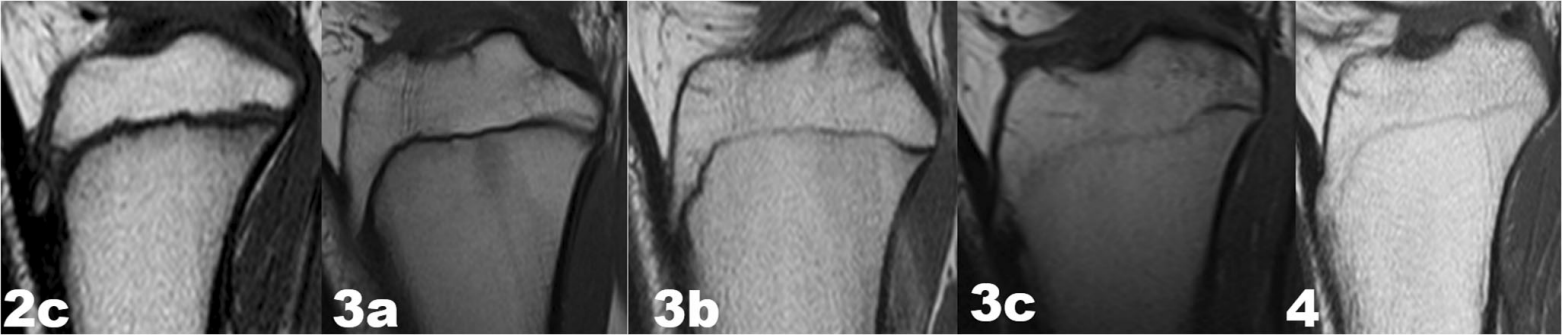
T1-weighted turbo spin echo (T1-TSE) sequences in the sagittal plane on MRI images: stages 2c,3a,3b,3c and 4 according to combined scoring system described by Schmeling et al. [21] and Kellinghaus et al. [22] for proximal tibial epiphysis

Estimation of the ossification stage of both epiphyses was possible in our sample. Figures 1 and 2 show the MRI findings for ossification stages 2c, 3a, 3b, 3c, and 4 that were observed for both epiphyses. The remaining ossification stages were not found within the study population.

In the distal femoral epiphysis, stage 2c was first noted at the age of 10.1 years, stage 3a at 12.8 years, stage 3b at 15.1 years, stage 3c at 14.6 years, and stage 4 at 15.4 years for females. For males, stages 2c, 3a, 3b, 3c and 4 were first observed at 10.0, 12.7, 15.1, 15.8, and 17.0 years, respectively.

In proximal tibial epiphysis, stage 2c was first noted at the age of 10.1 years, stage 3a at 11.8 years, stage 3b at 13.0 years, stage 3c at 14.0, and stage 4 at 15.1 years for females. For males, stages 2c, 3a, 3b, 3c, and 4 were first observed at 10.0, 12.7, 13,7, 15.1, and 15.8 years, respectively.

Tables 3 and 4 show the minimum and maximum ages at which the stages were noted, with lower and upper quartiles, medians, and means and standard deviations of all parameters.

**Table 3:** Minimum and maximum ages, with means ± SDs, lower and upper quartiles and medians, at all stages of distal femoral epiphysis

Stage	Sex	N	Mean ± SD	Min-Max	LQ; UQ;Median
2c	Female	34	11.21 ± 0.82	10.1–12.9	10.625;11.10;11.80
	Male	36	12.35 ± 1.53	10.0–15.3	10.90;12.95;13.40
3a	Female	28	13.91 ± 0.91	12.8–15.7	13.30;13.60;14.60
	Male	44	15.65 ± 1.41	12.7–18.7	15.20;15.60;16.40
3b	Female	8	15.32 ± 0.30	15.1–15.8	15.10;15.20;15.675
	Male	14	16.52 ± 0.78	15.1–17.5	16.10;16.70;17.30
3c	Female	26	16.27 ± 1.22	14.6–18.8	15.25;16.30;17.25
	Male	36	17.26 ± 1.42	15.8–21.9	16.50;16.90;17.80
4	Female	218	23.63 ± 3.96	15.4–29.8	20.175;24.20;26.90
	Male	205	23.91 ± 3.19	17.0–29.8	21.30;24.00;26.65

**Table 4:** Minimum and maximum ages, with means ± SDs, lower and upper quartiles and medians, at all stages of proximal tibial epiphysis

Stage	Sex	N	Mean ± SD	Min-Max	LQ; UQ;Median
2c	Female	30	11.07 ± 0.74	10.1–12.9	10.625;11.10;11.80
	Male	26	11.90 ± 1.48	10.0–13.7	10.90;12.95;13.40
3a	Female	16	13.12 ± 0.75	11.8–14.6	13.30;13.60;14.60
	Male	38	14.82 ± 1.57	12.7–18.5	15.20;15.60;16.40
3b	Female	8	13.55 ± 0.41	13.0–14.0	15.10;15.20;15.675
	Male	2	16.20 ± 3.5	13.7–18.7	16.10;16.70;17.30
3c	Female	20	15.15 ± 0.69	14.0–16.4	15.25;16.30;17.25
	Male	20	15.86 ± 0.52	15.1–16.6	16.50;16.90;17.80
4	Female	240	22.97 ± 4.31	15.1–29.8	20.175;24.20;26.90
	Male	249	22.73 ± 3.89	15.8–29.8	21.30;24.00;26.65

## Discussion

The presented results show that the minimal ages of patients with stage 4 ossification were 15.1 years for females and 15.8 years for males for the distal tibial epiphysis. Regarding the distal femoral epiphysis, a minimum age of 15.4 years for females and 17 years for males were obtained, which provide significant new insights in age estimation in general T1-TSE sequence MRI using the Schmeling and Kellinghaus staging method.

Our results on the age of first occurrence of the different ossification stages are similar to those from the studies of Kramer et al. [14, 15], Fan et al. [17], and Ottow et al. [16]. The results of all studies are presented in Table 5.

**Table 5:** The comparison of the minimum ages of the present study with previous T1-weighted turbo spin echo (T1-TSE) sequence MRI studies

Study	Nationality	Study protocol	Proximal tibial epiphysis			Distal femoral epiphysis		
			Stage	Female	Male	Stage	Female	Male
Krämer et al [14, 15]	Germany	3.0-T MRI T1-weighted turbo spin echo (T1-TSE) sequence in sagittal orientation	2c	10.1	10.1	2c	10.1	10.1
			3a	11.4	12.2	3a	11.4	12.2
			3b	–	13.9	3b		15.0
			3c	14.3	15.0	3c	15.6	15.0
			4	15.6	16.3	4	16.2	18.3
Ottow et al [16]	Germany	3.0-T MRI T1-weighted turbo spin echo (T1-TSE) sequence in coronal orientation	2c	12.11	12.13	2c	12.11	12.05
			3a	12.74	12.05	3a	13.39	13.68
			3b	13.39	15.18	3b	14.73	–
			3c	13.85	15.80	3c	14.53	16.13
			4	15.87	17.46	4	16.13	17.46
Fan et al [17]	China	1.5-T MRI T1-weighted turbo spin echo (T1-TSE) sequence in sagittal orientation						
			2c	11.00	11.00	2c	11.00	11.00
			3a	11.40	12.24	3a	11.40	12.20
			3b	13.80	15.20	3b	13.80	16.20
			3c	13.80	14.50	3c	14.10	14.50
			4	14.80	15.90	4	14.70	16.90
Present study	Turkey	1.5-T MRI T1-weighted turbo spin echo (T1-TSE) sequence in sagittal orientation	2c	10.1	10.0	2c	10.1	10.0
			3a	11.8	12.7	3a	12.8	12.7
			3b	13.0	13.7	3b	15.1	15.1
			3c	14.0	15.1	3c	14.6	15.8
			4	15.1	15.8	4	15.4	17.0

Nonclinical indications—such as for forensic purposes—involve drawbacks, especially in terms of children’s rights and have been addressed by many organizations dealing with children’s rights and radiation safety, emphasizing that nonionic methods should be used instead [9–11]. Hence, the obtained data in this research project will help to move forward the MRI-based age estimation approach on a global perspective.

In the presented study, very low intra- and inter-observer errors show the consistency of the study itself; it is also compatible with the other studies [13–16]. Although our study was carried out by a group of researchers with methodologically high experience, the effect of method-specific experience limitations on each observer, which was also emphasized by Wittschieber et al. [27], should be considered in future studies.

The results in this study that comparison of the male and female data revealed statistically significant differences. These data generally support the importance of sex discrimination for the distal femoral and proximal tibial epiphyses. Ottow et al. [16] demonstrated some remarkable differences in this regard. The differences may have therefore occurred due to an unbalanced distribution for each age group and sex (Table 5).

Studies on age estimation use different staging systems and MRI-specific measurement techniques. This situation can be thought to arise from the desire to define the most appropriate, most reproducible, and useful method for age estimation by the various researchers. This observation is also remarkable in studies on the MRI analysis of the distal femoral epiphysis and proximal tibial epiphysis [12–19]. All studies, except Jopp et al. [19], used the Schmeling staging system and Kellinghaus’s substages in the evaluation of the distal femoral epiphysis and proximal tibial epiphysis with T1- TSE sequence MRI. The Schmeling and Kellinghaus staging systems make use of a bone biology-based staging system, and its reproducibility and feasibility on X-ray and CT images, as well as MRI with T1 sequences, have been shown on various research projects [14–17, 23–25]. Verified data on the trabecular architecture can be obtained with the T1- TSE sequence [28, 29].

In the current literature on age estimation with MRI, combined methods have also been tested. Vieth et al. [18], who used a 3.0 T MRI scanner on the knee joint and acquired T1-TSE and a T2-weighted TSE sequences with fat suppression by spectral presaturation with inversion recovery (SPIR). A new staging system has been defined by the combination of data obtained with these two different sequences. With this combined method, except for the proximal tibial epiphysis in females, the minimal ages of all stage 6 are over 18 years old, and the Vieth method provides important data for this critical age definition.

Another factor that comes to mind when comparing MRI analyses that different strength of the magnetic field of MRI. Saint-Martin et al. [20] argued that magnetic field had not effect. However, there is no comparative study showing the effect of magnetic field differences on the data.

Although the studies did not include the socioeconomic data of the populations, any comparisons of the differences in these data should take into account the different populations used in all compared studies. According to the human development index published by the United Nations in 2019, Turkey is ranked 59th; China, 85th; and Germany, 4th [30]. It would be useful to consider these difference in the evaluation of the results [31, 32].

Most past age estimation studies were conducted retrospectively and with unbalanced distribution, and socioeconomic data were not presented in these studies. The fact that 649 out of 406 (ca. 65.5%) of our cohort are in the age bounds above 18 years of life, it might also indicate a selection bias. This fact is relevant since this lowers the chance to document the lower and upper extremes of the stages, which is a common limiting factor in age estimation studies and should be kept in mind. Balanced age distribution in prospective studies could important for demonstrating the applicability of the method [16, 18, 23].

Limited access to scanners, high costs, and long MRI examination times might be a limiting factor of using the MRI in forensic purposes. Nonetheless, these limitations can be justified due to the low number of patients for age estimation cases in general and the varying level of urgency for each case.

This study contributes to increase the comparable population database on a global scale, supporting T1-TSE MRI as a noninvasive method and the applicability and reproducibility of the Schmeling and Kellinghaus staging method. Future multicenter studies, including the combination of different MRI sequences and staging system as in the studies of Vieth et al. [18], are needed to achieve for more detailed information on knee joint for assessment of forensic age.
